# The European Union Current Asylum Policy: Selected Problems in the Shadow of COVID-19

**DOI:** 10.1007/s11196-020-09744-3

**Published:** 2020-07-02

**Authors:** Anna Doliwa-Klepacka, Mieczysława Zdanowicz

**Affiliations:** Faculty of Law, University of Bialystok, ul. Mickiewicza 1, 15-213 Bialystok, Poland

**Keywords:** Migration crisis, COVID-19, Hate speech, Temporary reintroduction of border controls

## Abstract

Recent years in Europe have generated situations requiring the European Union to take extra-coordinated action in the field of asylum policy. The sudden and growing influx of refugees to Europe in 2015 and 2016 has caused the collapse of the previous common European asylum system. The European Union has taken a number of measures to resolve this crisis situation. When the situation seemed to be under control, a new challenge emerged in early 2020. The first COVID-19 infectious disease case was reported in Europe, and on 13 March 2020 the WHO reported that Europe had become the epicentre of the coronavirus pandemic. The measures taken by individual countries and the European Union to limit the spread of the virus have had a significant impact on many spheres of state and individual functioning, including the situation of persons seeking international protection. This publication consists of three parts. The first part discusses actions taken by the European Union in the face of the migration and refugee crisis that emerged in 2015 and 2016. The second part presents one of the limitations introduced in connection with preventing the spread of COVID-19, which has a huge impact on persons wishing to seek international protection, i.e., changes in the regime of crossing borders and entering the territory of particular countries. The third one points out selected problems experienced by persons seeking protection who already stay in the territory of EU Member States.

## Introduction

Recent years in Europe have generated situations requiring the European Union to take extra-coordinated actions in the field of asylum policy. The sudden and growing inflow of refugees into Europe in 2015 and 2016 has led to the collapse of the common European asylum system based on the Dublin III Regulation determining the state responsible for examining an asylum application [37].

In 2015, as many as 1,255,600 people submitted applications for refugee status or another form of protection in European Union countries. This was a significant increase of 123% compared to 2014. Applicants were mainly from countries in the Middle East and Africa: Syrian citizens (362,800 people), whose number doubled, Afghan citizens (178,200 people), whose number almost quadrupled, and Iranians (121,500 people), whose number increased seven times compared to 2014. Applications by nationals of these countries accounted for more than half of all asylum applications and were lodged mainly in Greece and Italy [21]. The European Union has taken a number of measures to address the crisis situation. When it seemed to be under control, a new challenge emerged in early 2020. The first COVID-19 infectious disease case was reported in Europe, and on 13 March 2020 the WHO reported that Europe had become the epicentre of the coronavirus pandemic. The measures taken by individual countries and the European Union to limit the spread of the virus have had a significant impact on many spheres of state and individual functioning, including the situation of persons seeking international protection.

This publication consists of three parts: the first one discusses actions taken by the European Union in the face of the migration and refugee crisis that emerged in 2015 and 2016; the second part presents one of the limitations introduced in connection with preventing the spread of COVID-19, which has a huge impact on persons wishing to seek international protection, i.e., changes in the regime of crossing borders and entering the territory of particular countries; the third one points out selected problems experienced by persons seeking protection who already stay in the territory of EU Member States.

The aims of the publication are: (1) to present solutions related to the relocation of refugees, which were supposed to be the main way of solving the asylum crisis and to assess the effectiveness of this mechanism, as well as to identify other solutions adopted in this respect; (2) to present temporary restrictions on border traffic justified by the COVID-19 pandemic and their impact on international personal traffic; (3) to present the impact of the COVID-19 pandemic on anti-immigration sentiment and the situation of migrants who are already on the European Union territory.

## The European Union and the Asylum Crisis

Establishing the Common European Asylum System, the European Union introduced responsibility criteria and mechanisms for the examination of an asylum application for one country. The first regulations adopted in this area were included in the Convention implementing the Schengen Agreement and the in the Dublin Convention. These were further specified in internal acts of the European Parliament and the Council commonly referred to as the Dublin II Regulation and amending it Dublin III Regulation. The criteria for determining the state responsible for examining the asylum application include: the principle of family unity, the issuance of a residence permit or visa, illegal border crossing/illegal stay or place of the legal entry [42]. In practice, the main, disproportionate burden of examining asylum applications fell on Greece and Italy. This has created vast disparities and has pushed the European asylum system into chaos. The already inefficient asylum system in Greece [42, p. 404] has failed completely.

The European Council, at its extraordinary meeting on 23 April 2015, decided, inter alia, to increase assistance to frontline countries and to consider the possibility of organising emergency relocation of migrants on a voluntary basis. In addition, it decided to establish the first voluntary EU-wide resettlement pilot project offering placements for persons eligible for protection [19].

In view of the low effectiveness of the action taken by Member States, on 25 and 26 June 2015, the European Council decided that 60,000 persons should be temporarily and exceptionally relocated within 2 years, with 40,000 persons being relocated from Italy and Greece. All Member States were to participate. In addition, the European Council addressed the issues of return, readmission, reintegration and cooperation with countries of origin and countries of transit [17].

The conclusions and statements undertaken by the European Council resulted in the adoption of the conclusions of the government representatives of the Member States gathered in the Council (dated 22 July 2015), which implied the obligation for states to resettle 20,000 persons in clear need of international protection through multilateral and national systems. The Annex to the conclusions contained distribution quotas to individual countries [10].

However, key importance should be attached to the two decisions taken by the Council in September 2015. The first one concerned the relocation of 40,000 applicants [11] and the second relocation of 120,000 applicants to Member States [12].

Council Decision 2015/1523 of 14 September 2015 provided for the relocation from Greece and Italy to other EU Member States of persons in clear need of international protection. Within 2 years, 40,000 people were to be addressed by such measures—24,000 from Italy and 16,000 from Greece (article 4 of the Decision). Member States were to indicate regularly, and at least every 3 months, the number of applicants who could be swiftly relocated to their territory (Article 5(2) of the Decision). The Decision therefore gave Member States the possibility to decide on the number of persons and the date on which they would be accepted.

Council Decision 2015/1601 of 22 September 2015 concerned the relocation of 120,000 applicants to other Member States. According to the commitments made, 15,600 immigrants from Italy and 50,400 from Greece were to be relocated in accordance with the Annexes to the Decision (Article 4(1) of the Decision). The remaining 54,000 persons were to be relocated proportionally to the figures in Annexes I and II (Article 4(1)(c) of the Decision).

Both Council decisions were adopted by qualified majority with the Czech Republic, Hungary, Romania and Slovakia voting against (Finland abstaining).

The evaluating report presented by the European Commission indicates that the rate of relocation showed a constant upward trend. Over 2300 relocations were carried out monthly. Most Member States made regular declarations and relocations. As underlined by the Commission, Malta, Latvia and Norway relocated all their allocations, Finland, Lithuania and Luxembourg were close to meeting their commitments to Greece. Malta and Finland performed best in relocating their allowances from Italy [38].

However, as the Commission pointed out in the report, Hungary and Poland were the only Member States which had not made a single relocation, while the Czech Republic had not made any new declarations since May 2016 and had not made any relocations since August 2016 [38].

This negative attitude of some EU Member States towards relocation led to proceedings before the Court of Justice in Luxembourg. Thus, the Slovak Republic (C-643/15) and the Republic of Hungary (C-647/15) brought actions for the annulment of the Council Decision (EU) 2015/1601 of 22 September 2015 establishing provisional measures in the area of international protection for the benefit of Italy and Greece. On 6 September 2017, the Court of Justice, sitting in Grand Chamber, delivered a judgment dismissing the actions [13].

On the other hand, despite repeated requests from the Commission and the presentation of a reasoned opinion on 26 July 2017, Poland failed to accept a single applicant under the relocation procedures provided for in Council Decisions 2015/1523 and 2015/1601. The failure resulted in the European Commission bringing an action before the Court of Justice on 21 December 2017. On 22 December 2017 the Commission brought similar actions against Hungary (C-718/17) and the Czech Republic (C-719/17). The Court decided to examine the cases jointly. In a judgment delivered on 2 April 2020 it concluded that Poland, as well as the Czech Republic and Hungary, violated the EU law by refusing to participate in the temporary refugee relocation mechanism. In the Court’s view, Member States cannot, in order to evade the implementation of this mechanism, invoke either their obligations to maintain public order and protect internal security or the alleged malfunctioning of the relocation mechanism [14].

As most immigrants were coming to Europe via Turkey, the EU cooperation with the Turkish government was inevitable [26, p. 11]. In accordance with the agreement signed in March 2016, “All new irregular migrants crossing from Turkey into Greek islands as from 20 March 2016 will be returned to Turkey”. In return, “For every Syrian being returned to Turkey from Greek islands, another Syrian will be resettled from Turkey to the EU” [18]. In addition, the EU has allocated 6 billion euro under the EU Facility for Refugees in Turkey. Dahl and Dziudzik emphasize that even if at first it seemed that the agreement would not bring about the expected results (the EU accused Turkey of violating human rights and Turkey criticized the EU for its sluggishness in implementing the agreements), eventually, the agreement helped to significantly reduce the inflow of migrants from Turkey to Europe [15, p. 19]. The Commission also positively evaluated the implementation of the Agreement, indicating that Estonia, Finland, Germany, Ireland, the Netherlands, Sweden and the United Kingdom, as well as three associated states (Iceland, Liechtenstein and Switzerland) have fully complied with their obligations regarding the resettlement programme [38].

Dahl and Dziudzik wrote that the issue of migrants arriving in Europe via Turkey could not been considered fully resolved. The authors pointed out that bilateral relations in the face of the ongoing political changes would make it difficult to maintain the agreement and the problem could occur again [15, p. 19]. And so it happened. In February 2020, the problem of refugees coming to the EU via Turkey returned, as Turkey opened its borders with Greece and Bulgaria.

In 2016, the European Commission proposed a reform of the EU’s asylum policy, which envisaged, among other things, a permanent system for the distribution of refugees, which would be activated automatically in a crisis situation, as well as the possibility of buying out of the obligation to relocate [35]. This proposal was criticized by the states, and in 2017 the European Parliament proposed a solution which, among other things, departs from the excessive burden on the country of first entry and introduces relocation based on a permanent corrective allocation system to the states that have the lowest percentage of admissions [20]. Mikołajczyk considers the Commission’s proposal to be restrictive, both for the Member States and for persons seeking international protection. However, according to the author, the amendments of the European Parliament greater refer to the principle of solidarity contained in Article 80 of the TFEU and take into account the rights of migrants to a greater extent [32, p. 9]. Capicchiano Young also believes that the Commission’s proposal exacerbates inequalities in the burden on Member States, mainly due to the abolition of financial safeguards for countries that are particularly vulnerable to the flows of refugees [2, p. 373].

On 3 February 2017, members of the European Council adopted the so-called Maltese Declaration aiming to limit migration from North Africa. The European Union recognised Libya as a key partner in this regard, committing itself to help Libya in establishing conditions to fight migrants, assist in the fight against illegal human traffickers and support the Libyan Border Guard [41, p. 473].

In May 2018 in Marrakech, at a conference co-organised by the European Commission, the Declaration was adopted setting out a programme to combat migration in the course of the next 2 years.

Despite the fact that since 2017 the scale of the inflow of foreigners to Europe has not been so intense, it is not possible to speak unambiguously about solving the asylum crisis.

## Border Crossing in the European Union During the COVID-19 Pandemic

The rules on crossing the borders of the EU Member States belong to common policies. The Schengen Borders Code (Code, SBC) is the basic act of the European Union law in this area [36]. The Code regulates, inter alia, clearance on persons at the external borders, entry conditions and the conditions for the temporary reintroduction of border controls at certain borders within the Schengen area (excluding clearance at the internal borders of 22 EU countries plus Iceland, Liechtenstein, Norway and Switzerland) [24, p. 163].

According to Title IV of the Code, in case of a serious threat to public policy or internal security in a Member State, that Member State may exceptionally reintroduce border control at all or certain sections of its internal borders. Article 25 of the SBC sets out general principles in this respect. Control may be reintroduced for a limited period of time, not exceeding 30 days, or for the time of the foreseeable existence of this serious threat if it exceeds 30 days. The scope and duration of the temporary reintroduction of border control at internal borders must be introduced in accordance with the principle of proportionality, only to the extent necessary to respond to the serious threat concerned. If the threat persists, the state may prolong border control for further periods of 30 days. The total period of reintroduction of border control at internal borders may not exceed 6 months. In case of exceptional circumstances, the total period may be extended up to a maximum of 2 years (Articles 25 and 29 of the SBC).

Thus, where there is a serious threat to public policy or internal security, border controls may be reintroduced by the threatened Schengen countries either on the basis of Article 27 of the Code for foreseeable events such as major sporting events, conferences, etc. (for a period of not more than 6 months), or on the basis of Article 28 of the Code in cases of events requiring immediate action (for a maximum of 2 months) [16].

The second option concerns the occurrence of exceptional circumstances threatening the overall functioning of an area without internal border control (Article 29 of the Code). The temporary reintroduction of internal border controls is possible when the Schengen evaluation mechanism (i.e., the system of cooperation between Member States and the European Commission) reveals that the overall functioning of the area is jeopardised by persistent serious deficiencies related to external border controls. In case of serious irregularities in the conduct of external border controls, the European Commission may recommend EU countries to take certain actions. As a last resort, in order to protect common interests on a proposal from the European Commission, the Council may recommend that one or more Member States decide to reintroduce border controls along the whole or specific sections of their internal borders (for a maximum of 2 years).

In the context of the COVID-19 pandemic, many coordination actions have been taken at European Union level. This publication refers to only some of them, relating to freedom of movement and procedures for migrants.

Already on 10 March 2020, members of the European Council stressed the need for a common European approach, close coordination with the European Commission and the development of common guidelines [9]. Referring to this position of the European Council and to the provisions of the Schengen Borders Code, the European Commission adopted a Communication (dated 16 March 2020) to the European Parliament, the European Council and the Council calling for a temporary restriction of non-essential travel to the EU due to COVID-19, for an initial period of 30 days [4]. It was agreed that the temporary restrictions would exclude, inter alia, persons in need of international protection and persons who have to be admitted to the territory of the Member States on other humanitarian grounds. Any potential restrictions on asylum, return and resettlement must be proportionate, applied without discrimination and must take into account the principle of non-refoulement [39].

In response to the epidemic risks associated with COVID-19, countries have introduced a number of restrictions, including those on border traffic. Their aim was to slow down the spread of the virus and flatten the disease curve, which was to protect national health care systems from inefficiencies. Based on the data provided by states, only 16 Member States of the European Union have introduced a state of emergency (Belgium, Cyprus, Czech Republic, Denmark, Estonia, Finland, France, Hungary, Italy, Latvia, Lithuania, Luxembourg, Malta, Romania, Slovakia, Spain) [31].

The Joint Statement of the Members of the European Council related to the COVID-19 pandemic was adopted on 26 March 2020 [28]. Members of the European Council, on the one hand, stressed the urgent need to combat the COVID-19 pandemic and its immediate consequences. On the other hand, they called for preparations to be made for a gradual return to normal functioning of the societies and economies of the European Union Member States.

On 30 March 2020, referring to previously presented positions and suggestions to Member States, the Commission provided guidance on how to implement temporary travel restrictions, how to facilitate repatriation from all over the world and how to deal with people who are forced to stay in the EU longer than allowed—due to travel restrictions [7]. On 8 April 2020, the Commission invited Schengen Member States and Schengen Associated States to prolong the temporary restriction on non-essential travel to the EU until 15 May [5]. All EU Member States (except Ireland) and non-EU Schengen countries have since taken national decisions to implement and prolong the travel restrictions. The travel restrictions do not apply to EU citizens, citizens of non-EU Schengen countries and their family members, and non-EU nationals who are long-term residents in the EU for the purpose of returning home. Member States should not apply the restrictions to specific categories of travellers with an essential function or need.

When looking for the relevant documents coordinating EU Member States’ approaches to COVID-19, it should also be noted that, in response to the European Council’s call of 26 March 2020, the Commission (in cooperation with the President of the European Council) developed Joint European Road map towards lifting COVID-19 containment measures [27]. The document was presented on 15 April 2020. The roadmap provided for the phasing out of the containment measures introduced due to COVID-19, including a coordinated approach to restoring freedom of movement and lifting internal border controls. The Roadmap is a non-binding act, providing a set of recommendations to national governments. On the other hand, in addition to a long-term plan to phase out the restrictive measures, the Commission has advocated an extension of the temporary travel restrictions that are not unnecessary. On 8 May 2020, the Commission invited Schengen Member States and Schengen Associated States to extend the temporary restrictions on non-essential travel to the EU for another 30 days, until 15 June 2020 [6].

In the Schengen area, governments have taken different approaches to contain the COVID-19 pandemic, also taking into account the economic impact of the restrictions. Analysing the data on international movement restrictions as of 20 April 2020, it should be noted that all European Union countries applied restrictions. 14 states introduced countrywide restrictions (Belgium, Cyprus, Denmark, Estonia, Finland, France, Greece, Luxembourg, Malta, Poland, Portugal, Slovakia, Slovenia, Spain). In others, the restrictions were partial or regional in nature [29]. It can be noted that the high level of restrictions does not always apply to the countries with the highest virus incidence rates. For instance, the international movement restrictions in the entire territory were applied Cyprus, Estonia, Finland, Greece, Malta, Poland, Slovakia, Slovenia—with less than 10,000 COVID-19 cases reported, while only regional movement restrictions were imposed in Italy or Germany where the number of cases reported exceeded 100,000. When we take a look at the flights restrictions, the situation is even more diverse. Two of the EU states had no restrictions in this field (Denmark, Ireland), while in 12 Member States the air passenger traffic was completely stopped (Croatia, Cyprus, Finland, France, Greece, Lithuania, Luxembourg, Malta, Poland, Slovakia, Slovenia, Spain) [29]. Partial restrictions have been introduced in the remaining 13 EU Member States.

According to data as of 11 May 2020 (already after a further extension of unnecessary travel restrictions was recommended by the Commission), some Member States have modified their approach in this respect [30]. The number of countries where international movement restrictions have been maintained with a nationwide reach has fallen to nine (still total restrictions have been maintained in Cyprus, Denmark, Estonia, Finland, Luxembourg, Malta, Poland, Portugal, Spain). In the others, the restrictions were partial/regional in nature.

Analysing the flights restrictions, it should be underlined that the number of countries that lifted restrictions in this respect has increased to three (Denmark, Estonia, Ireland), noting that each of these countries has retained general restrictions on international traffic. The total suspension of flights was maintained in 11 EU countries (Croatia, Cyprus, Finland, Lithuania, Luxembourg, Malta, Poland, Romania, Slovakia, Slovenia, Spain). In the remaining states, the restrictions were partial/regional in nature [30].

On 13 May 2020 the Commission presented a further package of guidelines for the progressive abolition of the introduced travel restrictions, including the Communication “COVID-19. Towards a phased and coordinated approach for restoring freedom of movement and lifting internal border controls” [8]. It proposed a gradual and coordinated approach to the lifting of previously introduced restrictions in Member States with sufficiently similar epidemiological situations. If the epidemiological situation so requires, certain restrictions may be reintroduced. The Commission proposed that Member States should base their action on three criteria. The basic ones are epidemiological criteria, in accordance with the guidelines of the European Centre for Disease Prevention and Control (ECDC). The second is the possibility of applying containment measures throughout the journey, including measures imposed at border crossing points. The third criterion refers to economic and social considerations (hence, inter alia, the priority of cross-border traffic in key business areas and for personal reasons) (Table 1).

**Table 1:** Current temporarily reintroduced border controls *Source:* own elaboration based on Commission data: https://ec.europa.eu/home-affairs/what-we-do/policies/borders-and-visas/schengen/reintroduction-border-control_en. Accessed 29 May 2020

Legal basis for the border control reintroduction	States	Period of reintroduced border control	Scope of reintroduced border control	Justification for the border control reintroduction
Temporarily reintroduced border controls in the context of *cases requiring immediate action* (Art 28 of the codified SBC)	Iceland	24 April–3 June 2020	At all internal borders	Coronavirus COVID-19
	Slovakia	8 April–27 May 2020	At all internal borders	Coronavirus COVID-19
Temporarily reintroduced border controls in the context of *foreseeable events* (Art 25 and 26 of the codified SBC)	Austria	8 May–15 June 2020	Land borders with Germany, Italy, Switzerland, Liechtenstein, Slovakia and Czechia	Coronavirus COVID-19
	Austria	12 May 2020–11 November 2020	Land borders with Hungary and with Slovenia	Secondary movements, risk related to terrorists and organized crime, situation at the external borders
	Belgium	19 May–8 June 2020	All internal borders	Coronavirus COVID-19
	Czechia	14 May–13 June 2020	Land borders with Austria and Germany, air borders	Coronavirus COVID-19
	Denmark	12 May–12 November 2020	All internal borders	Coronavirus COVID-19 (to the extent necessary), terrorist threats, organized criminality
	Estonia	18 May–16 June 2020	Internal air and sea borders	Coronavirus COVID-19
	Finland	19 March–14 June 2020	All internal borders	Coronavirus COVID-19
	France	1 May–31 October 2020	All internal borders	Coronavirus COVID-19; continuous terrorist threat and risk of terrorists using the vulnerability of States due to COVID-19 pandemics; support to measures aiming at containing the spread of virus
	Germany	16 May–15 June 2020	Land and air borders with Austria, Switzerland, France, Denmark, Italy and Spain, sea border with Denmark	Coronavirus COVID-19
	Germany	12 May–11 November 2020	Land border with Austria	Secondary movements, situation at the external borders
	Hungary	12 May–11 November 2020	All internal land and air borders	Coronavirus COVID-19
	Lithuania	14 May–31 May 2020	All internal borders	Coronavirus COVID-19
	Norway	12 May–11 November 2020	Ports with ferry connections with Denmark, Germany and Sweden	Terrorist threats, secondary movements
	Norway	15 May–13 August 2020	All internal borders	Coronavirus COVID-19
	Poland	14 March–12 June 2020	Land borders with Czechia, Slovakia, Germany, Lithuania, sea borders, air borders	Coronavirus COVID-19
	Portugal	15 May–15 June 2020	Land border with Spain	Coronavirus COVID-19
	Slovakia	28 May–26 June 2020	At all internal borders	Coronavirus COVID-19
	Spain	10 May–7 June 2020	All internal borders	Coronavirus COVID-19
	Sweden	12 May–11 November 2020	To be determined but may concern all internal borders	Terrorist threats, shortcomings at the external borders
	Switzerland	14 May–8 June 2020	All internal air and land borders except from borders with Liechtenstein	Coronavirus COVID-19

At this stage of the COVID-19 epidemic, Member States have invoked two justifications when notifying the European Commission of the temporary reintroduction of internal border controls: cases requiring immediate action (Art 28 of the codified SBC) and cases where exceptional circumstances put the overall functioning of the Schengen area at risk (Article 29 of the codified SBC) [1]. Almost all Schengen States have explicitly or implicitly invoked the COVID-19 epidemic threat (only Sweden and Norway have generally invoked the terrorist threat).

With regard to data on immigration flows into the European Union [22] in the COVID-19 emergency, the Mediterranean remains the dominant route [3]. Between January and April 2020, 11,211 persons came to the EU illegally via the Eastern Mediterranean Route (Turkey sea route), with the highest number of arrivals from Afghanistan (3250 people), Syria (2446 people), Turkey (940 people), Somalia (538 people) and Pakistan (446 people).

Another direction of illegal immigration is the Western Balkans. Between January and April 2020, 5987 immigrants entered the EU from there, mainly from Syria (4002), Afghanistan (977) and Iraq (219).

The third is the Central Mediterranean route (via Libya). In the same period, 4064 illegal immigrants entered through this route and the top countries of origin in this section include: Bangladesh (654), Sudan (598), Côte d’Ivoire (445), Somalia (347) and Algeria (319).

At the same time, 3016 people made it through the Western Mediterranean route (via Morocco), the largest number of whom came from Algeria (1223), Unspecified sub-Saharan nationals (1075) and Morocco (632).

The fifth place is assigned to illegal crossings through the EU’s eastern land border (border with Poland). Here, 120 cases were detected between January and April 2020. Majority of persons came from: Turkey (30), Bangladesh (13), Vietnam (12), Ukraine (8) and Algeria (6).

The impact of COVID-19 on migration is clearly seen when comparing the data for April 2020 with data for March 2020 [23]. In April 2020, the total number of detected illegal border crossings along the main European migration routes fell by as much as 85% (to around 900) compared to March 2020, the lowest figure since Frontex started collecting border data in 2009. At the time of the submission of the article, the full data were not yet known, inter alia, due to delays in the transmission of border crossing data by national authorities. The total number of illegal border crossings in the first 4 months of 2020 was about 26,650, roughly the same as in the same period last year. The record low figure was undoubtedly due to the impact of the COVID-19 epidemic.

In every case, the decrease compared to March 2020 was very visible: Eastern Mediterranean route—down as much as 99%, Western Balkans—down 94%, Western Mediterranean—down 82%, Central Mediterranean—down 29%. When we compare the data for January–April 2020 with the same period in 2019, the results are different [22]. The largest fall in illegal overruns year-on-year was observed on the Western Mediterranean routing—a fall of 53%. The largest Eastern Mediterranean route today also saw a decrease of 18%. On the next two of the largest routes, the first 4 months of 2020 brought an increase compared to the same period in 2019: Central Mediterranean route—an increase of 331%, Western Balkans migratory route—an increase of 60%.

## Situation of Migrants Already Staying in the European Union Countries

Regardless of the difficulties introduced in the mere crossing of the borders of the Schengen Member States, the widespread threat of an “invisible enemy” and the multi-faceted epidemic restrictions of COVID-19 affect migrants, including refugees, in virtually every aspect of life. The brutalisation of everyday life is clearly escalating in Greek refugee camps. According to media reports, especially at night, the camps are turning into a real war zones. With such a high population density and widespread shortages, there is a constant struggle for survival, food, doctor’s appointment, etc., every person is a potential enemy [25].

The first cases of COVID-19 infection appeared in the Greek refugee camp Ritsona (north of Athens) in the first days of April. The camp, which is home to almost 3000 people, was cut off from the world overnight—the entrances were secured by the police, with no possibility of leaving the area. A few days later, a similar situation occurred in another camp in the area—Malakasa with 1600 people inside. At the end of April, infections with the virus appeared in hotel for asylum seekers located in the town of Kranidi in the Peloponnese. The two-week quarantine of the refugee centres turned into isolation for over a month [25].

As of mid-May 2020, there are no official data on incidents in refugee camps on the Greek islands in the Aegean Sea, near Turkey. There are about 42 thousand people there, with more than 20 thousand in Moria, Lesbos alone. However, taking into account the general situation and the lack of testing, the outbreak of the pandemic seems only a matter of time.

According to data from the Polish Rescue Foundation (Fundacja Ocalenie [40]), in Poland, in spite of closing the borders and limiting traffic to an absolute minimum, the deportations were not stopped. Despite the limitations of the pandemic the deportations are still carried out, especially in Chechnya.

According to the reports, the Border Guard informs about the decision the night before deportation, which violates the fundamental right to defence and legal aid. Despite the closure of the borders, deportations are carried out by car to the Kaliningrad Region, from where, after the quarantine, the foreigner is transported through Moscow to Chechnya. There are no official data of the Border Guard on such cases, however, according to the information provided by the Rescue Foundation, these are not isolated cases since the outbreak of the epidemic in Poland [33].

It should be noted that the provisions of the so-called Anti-Crisis Shield in Poland contain regulations concerning foreigners, including: extension of legal residence and work permits, extension of application deadlines, as well as postponement of deadlines for foreigners to leave the territory of Poland and voluntary return specified in decisions obliging a foreigner to return. However, the problem is that they only apply to persons who have received a decision to commit to return during the course of a state of epidemiological emergency or during the epidemiological state, and not to those who have received them before.

The period of pandemic is conducive to disinformation and unrest, which results in an increase in hate speech, including in relation to refugees. The Association “Never Again” (founded in 1996, an independent, apolitical, expert NGO) published a report which documents acts of racism, xenophobia and discrimination that occurred in the context of COVID-19 in Poland. For example, on 2 April 2020, the Polish public television channel (TVP1) published material accusing refugees staying in camps in Greece of spreading the COVID-19. The information concerned the judgment of the Court of Justice of the European Union of 2 April 2020 on the failure of Poland, the Czech Republic and Hungary to comply with their obligations under EU law in relation to the failure to relocate refugees. The co-author of the reportage claimed “Coronavirus was detected in a Greek refugee camp. As many as 20 immigrants from the Middle East are infected. […] Local residents are full of fear, because there is no shortage of escapes, and the situation in Greece is getting worse. But the CJEU judgment leaves no illusions: the safety of the residents of the community is less important than EU regulations” [34].

In the face of the emerging acts of hostility that have intensified in connection with the COVID-19 epidemic, the President of the city of Poznan in Poland appealed to support each other and not to provoke discriminatory behaviour. Under the content of the appeal, published, among others, on the epoznan.pl portal, various discriminating comments appeared, including, for example, “Out with foreigners!!!” [34].

The COVID-19 epidemic caused fear and uncertainty. These feelings in turn progressed into dangerous hostility in some people, which manifested itself in a hate speech addressed to foreigners, towards refugees.

## Conclusions

The recent period has been full of challenges for the implementation of asylum policy in the European Union. The years 2015–2016 showed a rapid inflow of foreigners to Europe, with overcrowded camps for applicants for international protection, especially in Greece and Italy. The main activities undertaken by the European Union focused on solving the problem of relocation. The Council took two decisions in this respect, the first (Council Decision 2015/1523) giving Member States the possibility to decide on the number of persons to be admitted and the deadline for their admission; the second (Council Decision 2015/1601) indicated the specific numbers of refugees to be admitted by each country. Although most Member States regularly carried out relocations, there were also those that questioned the decisions taken. The Court of Justice of the European Union, in its judgment of 6 September 2017, dismissed the action of Hungary and Slovakia for the annulment of Decision 2015/1601, while in its judgment of 2 April 2020, the Court stated that Poland, the Czech Republic and Hungary, by refusing to participate in the temporary refugee relocation mechanism, violated EU law. The judgment arrived when the decision was no longer valid. This creates an exceptional situation where a judgment confirms a breach by Member States and the act which gave rise to the obligations is no longer in force.

Another important instrument adopted at that time, which had a positive impact on reducing the number of foreigners coming to Europe, is the agreement signed between the EU and Turkey. It should also be noted that the European Commission has initiated work on the adoption of a permanent refugee distribution system that would be activated automatically in a crisis situation, but no binding legislation has been adopted to date.

The outbreak of the COVID-19 pandemic in Europe has undoubtedly not contributed to the improvement of the situation in the ongoing migration crisis. The temporary closure of the internal borders of the Member States of the European Union and the reintroduction of border controls have aggravated the problem, which has for years been mainly faced by Greece and Italy. On the other hand, the feelings of danger in individual countries have led to tensions and radicalisation of behaviour towards another person, especially if he or she is different, a stranger.

This is undeniably a difficult time for people seeking international protection in Europe. The long period of stay in overcrowded camps waiting for relocation, closed borders, fear of the COVID-19 spread in large groups of people living in centres for asylum seekers, and finally the spreading hate speech—these are the phenomena that overshadow the standards of protection of the rights of beneficiaries of international protection developed over the years.
